# Toward Immersive Diabetes Education: Understanding User Needs for Virtual Reality–Assisted Self‐Management Support in Adults With Type 2 Diabetes Mellitus

**DOI:** 10.1155/nrp/4385505

**Published:** 2026-11-06

**Authors:** Nuurhidayat Jafar, Andina Setyawati, Sam Chidi Ibeneme, Akbar Akbar, Husnul Hatima Ahmad, Suriani Suriani

**Affiliations:** ^1^ Community Nursing Department Faculty of Nursing Hasanuddin University Jl Perintis Kemerdekaan Km 10, Tamalanrea Makassar 90245 Indonesia unhas.ac.id; ^2^ Medical and Surgical Nursing Department Faculty of Nursing Hasanuddin University Jl Perintis Kemerdekaan Km 10, Tamalanrea Makassar 90245 Indonesia unhas.ac.id; ^3^ Department of Physiotherapy David Umahi Federal University of Health Sciences Uburu Ebonyi Nigeria; ^4^ Faculty of Nursing Hasanuddin University Jl Perintis Kemerdekaan Km 10, Tamalanrea Makassar 90245 Indonesia unhas.ac.id

**Keywords:** diabetes self-management, digital health, qualitative research, Type 2 diabetes mellitus, user-centered design, virtual reality

## Abstract

Effective self‐management in Type 2 diabetes mellitus (T2DM) requires sustained adherence to diet, physical activity, medication, and lifestyle modification. However, conventional diabetes education often remains generic, one‐directional, and insufficiently responsive to the psychological, behavioral, and contextual challenges patients face in daily life. Immersive technologies such as virtual reality (VR) may offer a more engaging approach to diabetes education, yet evidence on the user needs that should inform such interventions remains limited. This study explored the psychological, behavioral, and educational needs of adults with T2DM and healthcare providers to inform the development of VR‐assisted self‐management support. A qualitative descriptive study was conducted involving adults with T2DM (*n* = 12) and healthcare providers (*n* = 6), purposively recruited based on their experience in diabetes self‐management or care; prior technology experience was not required. Data were collected through in‐depth interviews and analyzed using thematic analysis. Seven interrelated themes were identified: (1) psychological experiences of living with and managing diabetes, (2) motivation and adherence, (3) adaptation of physical activity, (4) dietary management challenges, (5) limitations of current diabetes education, (6) educational needs supporting self‐management, and (7) VR as an alternative educational medium. Participants described emotional distress, uncertainty, adherence fatigue, and difficulty translating advice into daily behaviors. Existing education was often perceived as overly general, medically framed, and insufficiently tailored to real‐life needs. Both patients and healthcare providers preferred education that is simple, interactive, practical, and grounded in everyday experiences. VR was viewed as a potentially valuable medium for simulating realistic self‐management scenarios, although concerns regarding usability and complexity were also raised. These findings provide a user‐centered qualitative foundation for developing immersive VR‐assisted diabetes self‐management support, intended to complement conventional education; its feasibility, sustainability, and effectiveness remain to be evaluated.

## 1. Introduction

Type 2 diabetes mellitus (T2DM) remains a major global public health challenge, with a rapidly increasing burden, particularly in low‐ and middle‐income countries. Recent estimates indicate that diabetes affects hundreds of millions of adults worldwide, with prevalence rising from 7% in 1990 to 14% in 2022 and disproportionately affecting resource‐limited settings [1]. Likewise, the International Diabetes Federation estimated that 536.6 million adults were living with diabetes in 2021, a figure projected to increase to 783.2 million by 2045 [2]. In Indonesia, diabetes prevalence has shown a persistent upward trend, increasing from 10.7% in 2013 to 11.8% in 2018 and remaining high at 11.3% in 2023, thereby placing increasing pressure on primary care and community‐based chronic disease management [3]. Beyond glycemic control, living with T2DM requires continuous engagement in complex self‐management behaviors, including dietary regulation, physical activity, medication adherence, and long‐term lifestyle modification.

Despite the central role of self‐management in diabetes care, sustained adherence remains difficult for many adults with T2DM. Evidence consistently shows that barriers to self‐management extend beyond knowledge deficits and involve psychological, behavioral, and contextual factors that shape how individuals respond to the daily demands of living with diabetes. These barriers include diabetes‐related distress, low self‐efficacy, frustration with the chronic and dynamic nature of the condition, and difficulties integrating recommended behaviors into everyday routines [4, 5]. Patients often report uncertainty in applying dietary and physical activity advice, competing work and family priorities, and challenges in translating clinical recommendations into practical decisions about eating, movement, and medication‐taking in real‐life settings. These difficulties are further influenced by health literacy, financial constraints, rural or geographically underserved residence, where geographic distance and limited access to healthcare services, diabetes education, self‐monitoring resources, and digital infrastructure may further complicate self‐management, as well as by family and social support and the broader social realities in which self‐management is enacted [6–8]. Although diabetes self‐management education (DSME) is recognized as an essential component of care, its implementation in routine practice often remains limited, and conventional educational approaches may still fail to adequately address patients’ psychosocial needs and lived experiences [9].

Traditional educational approaches such as verbal counseling, brief consultations, and printed materials have long been used to improve diabetes knowledge and support behavior change [10]. However, these approaches often remain generic, one‐directional, and insufficiently responsive to patients’ practical realities, preferences, and perceived needs [11]. Although they may increase awareness, they do not always support the sustained enactment of self‐management behaviors in real‐world settings, particularly when counseling is inconsistently delivered or lacks ongoing reinforcement [12, 13]. This limitation is especially relevant in chronic disease care, where effective education must be not only informative but also engaging, practical, and behaviorally meaningful. In Indonesia, a scoping review of the community‐based chronic disease management program (Prolanis) in primary health centers similarly reported limited patient education and inconsistent counselling, educational materials that were not tailored to cultural norms, literacy levels, or learning styles, and staff time constraints that limited the time available for education [14].

Digital health innovations offer important opportunities to strengthen diabetes education and self‐management support, particularly by extending access to educational resources and ongoing support beyond conventional clinic‐based encounters [15, 16]. Among these approaches, virtual reality (VR) represents a promising immersive modality with the potential to provide more engaging, interactive, and context‐rich learning experiences. Compared with static educational formats, immersive and digitally supported interventions may better facilitate experiential learning and more active engagement with self‐management tasks, including dietary decision‐making and lifestyle‐related behavior rehearsal [17]. Nevertheless, the benefits of digital approaches depend on users’ digital health literacy and access, which vary considerably across populations, and limited digital familiarity may hinder engagement with technology‐based education among adults with diabetes [18, 19]. In Indonesia, national policy is advancing digital health transformation in primary care, although the national blueprint does not specifically address immersive technologies such as VR [20].

Digital technologies have been widely investigated in diabetes care, including mobile health applications, virtual coaching, and web‐based self‐management programs [15–17, 21, 22]. However, this evidence mostly concerns the availability and effectiveness of digital tools, with less attention to what adults with T2DM and their healthcare providers need before such tools are designed. This gap is particularly evident for immersive technologies: reviews note that VR in diabetes remains at an early stage, with limited stakeholder input and uncertain applicability in low‐resource settings [23], and only 10 eligible studies of VR‐based self‐management for T2DM have been identified [24]. Although immersive and avatar‐based diabetes education has been piloted in other health‐system contexts [25, 26], little is known about the needs that should shape VR‐assisted diabetes education in Indonesian primary care. The effectiveness of digital support also depends on relevance, usability, and fit with users’ daily routines and capacity for sustained engagement [21, 22]. Therefore, rather than evaluating an existing technology, this study explores end‐user needs and expectations to inform a future VR‐assisted educational intervention.

Understanding user needs is therefore essential before developing immersive educational tools. A user‐centered approach ensures that digital interventions reflect users’ characteristics and real‐world contexts, whereas misalignment may lead to poor adoption; co‐design and participatory approaches enhance relevance, usability, and sustainability [27]. Human‐centered design and usability are likewise critical for engagement and implementation across diverse populations [28]. This study aimed to explore the psychological, behavioral, and educational needs of adults with T2DM and healthcare providers in order to inform the development of VR‐assisted self‐management support. The envisioned intervention is a scenario‐based, provider‐guided immersive program, with monitoring of use, delivered through a standalone head‐mounted display (a VR headset that operates without a computer or external sensors) alongside conventional diabetes education. Its proximal targets are patients’ understanding of diet, physical activity, and medication routines and their self‐efficacy in applying this knowledge [4], whereas self‐management behaviors, glycemic control, and diabetes‐related distress are longer‐term outcomes for future evaluation.

## 2. Methods

### 2.1. Study Design

This study employed a qualitative descriptive design, an approach well suited to generating straightforward, practice‐relevant descriptions of participants’ experiences and perspectives in health research [29]. This design was considered appropriate for exploring the psychological, behavioral, and educational needs of adults with T2DM and healthcare providers in order to inform the development of a VR‐assisted educational intervention.

### 2.2. Setting

The study was conducted in primary health care settings at community health centers (Puskesmas) in Jeneponto District, South Sulawesi, Indonesia, involving both adults with T2DM and healthcare providers (nurses and one physician) involved in diabetes care. These settings represent the frontline of community‐based diabetes management, where education and self‐management support are routinely delivered through outpatient services and health promotion activities. Data collection was supported by the Faculty of Nursing, Universitas Hasanuddin, which facilitated coordination and research implementation.

### 2.3. Participants and Recruitment

Participants included adults diagnosed with T2DM and healthcare providers involved in diabetes care and education. Participants were recruited using purposive sampling based on their relevance to the study objectives. Inclusion criteria for patients were: Age ≥ 18 years, diagnosis of T2DM, and ability to communicate experiences related to self‐management. Healthcare providers were eligible if they had experience in delivering diabetes care or education in clinical or community settings. A total of 18 participants were included, comprising 12 adults with T2DM and 6 healthcare providers. Recruitment continued until sufficient depth and richness of data were achieved. Prior experience with digital technology or VR was not an eligibility criterion. Because the study aimed to identify educational needs and expectations for a technology that participants had not yet used, adults with varying levels of digital familiarity were considered relevant, and including those with limited familiarity is necessary to identify accessibility and usability barriers, consistent with calls to involve a wide range of stakeholders in shaping VR tools for diabetes [23]. For healthcare providers, the criterion focused on experience in diabetes care or education, as their input concerned educational practice and the feasibility of delivering education in primary care rather than technical expertise. Participants’ digital familiarity is described in Table 1 and was considered when interpreting the findings.

**TABLE 1:** Characteristics of study participants.

Participant ID	Participant group	Age (years)	Sex	Occupation/role	Duration of T2DM/professional experience	Additional characteristics
P1	Adult with T2DM	36	Female	Honorary	5 years	Receives education via lectures, leaflets, brochures; uses digital media
P2	Adult with T2DM	46	Female	Teacher	5 years	Receives education via lectures and apps; limited digital use
P3	Adult with T2DM	26	Female	Housewife	5 years	Receives education via lectures and printed materials
P4	Adult with T2DM	45	Male	Farmer	6 years	Receives education via lectures; limited digital use
P5	Adult with T2DM	45	Female	Housewife	4 years	Receives education via lectures only
P6	Adult with T2DM	49	Female	Housewife	10 years	Receives education via lectures and apps
P7	Adult with T2DM	54	Female	Housewife	3 years	Receives education via lectures and apps
P8	Adult with T2DM	50	Male	Music player	8 years	Receives education via lectures; no digital use
P9	Adult with T2DM	52	Female	Housewife	3 years	Receives education via lectures; no digital use
P10	Adult with T2DM	52	Male	Civil servants	8 years	Receives education via lectures and apps
P11	Adult with T2DM	48	Female	Housewife	2 years	Receives education via lectures only
P12	Adult with T2DM	49	Female	Housewife	7 years	No structured education; no digital use
P13	Healthcare provider	48	Male	Doctor	> 5 years	Primary care physician; no digital education experience
P14	Healthcare provider	39	Female	Nurse	> 5 years	Experience in primary care; uses digital media
P15	Healthcare provider	39	Female	Nurse	> 5 years	Experience in diabetes care; uses digital media
P16	Healthcare provider	26	Female	Nurse	3 years	Early‐career nurse; no digital education experience
P17	Healthcare provider	27	Female	Nurse	1 year	Junior nurse; no digital education experience
P18	Healthcare provider	41	Female	Nurse	> 5 years	Experienced nurse; no digital education experience

### 2.4. Data Collection

Data were collected through semistructured, in‐depth interviews using separate interview guides for adults with T2DM and healthcare providers, developed based on the literature on diabetes self‐management, patient education, and digital health interventions. Interviews explored lived experiences of diabetes, psychological and behavioral challenges, self‐management practices, experiences with existing education, unmet educational needs, and expectations for digital and immersive learning approaches. For adults with T2DM, predefined probes explored practical challenges related to food portions, food choices, meal timing, social eating situations, and physical activity. Participants were also asked about the perceived usefulness of visual or simulated representations of food, portion sizes, and physical activity, followed by open‐ended questions regarding their expectations and preferred features for future VR‐based diabetes education. Healthcare providers were asked about common educational challenges, limitations of existing educational media, important content for VR‐based education, and features required for its effective and safe use. The complete interview guides are provided in Supporting Table S1. Interviews were conducted in Bahasa Indonesia, lasted approximately 30–60 min, and were audio‐recorded with participants’ consent. Field notes were also taken to capture contextual information and nonverbal cues. VR was introduced to participants through a brief verbal explanation of what it is and how it might be used for diabetes education; no headset or VR content was demonstrated. Participants’ responses therefore reflect their expectations and perceived needs in relation to the concept of VR, not experience of using it.

### 2.5. Data Analysis

Data were analyzed using thematic analysis with an inductive approach. Transcripts were read repeatedly to achieve familiarity, followed by coding, identification of patterns, and development of themes. Coding and theme development were conducted iteratively, with regular discussions among the research team to refine interpretations and ensure analytic rigor. The analysis focused on identifying key domains relevant to intervention development, including psychological needs, behavioral challenges, educational gaps, and user expectations for immersive diabetes education.

### 2.6. Rigor

Rigor was addressed using the trustworthiness criteria of Lincoln and Guba [30–32]: credibility, transferability, dependability, and confirmability. Credibility was supported by prolonged engagement with the data, an interviewer trained and experienced in qualitative research, verbatim transcription, coding and theme development by more than one researcher with iterative discussion, peer debriefing, and member checking. Transferability was supported by purposive sampling and a detailed description of the setting (Puskesmas in Jeneponto District), participants (Table 1), and illustrative quotations. Dependability was addressed by documenting data collection and analytic procedures, including the interview guides (Supporting Table S1), field notes, and team discussions. Confirmability was supported by an audit trail of analytic decisions, reflexive consideration of potential researcher bias, and quotations linking interpretations to the data.

### 2.7. Ethical Considerations

Ethical approval for this study was obtained from the Health Research Ethics Committee, Faculty of Nursing, Universitas Hasanuddin (Approval No. 039/UN4.18.3/TP.01.02/2026, dated 13 January 2026). The study was reviewed under an expedited review process and approved for implementation within the specified period.

All participants were informed about the purpose, procedures, and voluntary nature of the study prior to participation. Written informed consent was obtained from all participants. Confidentiality and anonymity were ensured throughout data collection, analysis, and reporting.

### 2.8. Use of Tools and Materials

The interview guides used in this study were developed by the research team and did not involve copyrighted instruments requiring permission for use.

## 3. Results

### 3.1. Participant Characteristics

A total of 18 participants were included in this study, comprising 12 adults with T2DM and 6 healthcare providers. As presented in Table 1, most adults with T2DM were female (75.0%), with ages ranging from 26 to 54 years and a mean age of 46.0 years. Most participants had lived with diabetes for more than 5 years, indicating substantial experience with long‐term disease management. Among healthcare providers, the majority were also female (83.3%), with ages ranging from 26 to 48 years and a mean age of 36.7 years. This group consisted predominantly of nurses, along with one physician, and most had more than 5 years of professional experience in primary care or diabetes‐related services. Overall, these characteristics indicate that the data were derived from participants with relevant lived and professional experience, thereby strengthening the depth and credibility of the findings. Familiarity with digital technology varied (Table 1). Five adults with T2DM reported using digital media or applications (one described this use as limited), one other described limited digital use, three reported no digital use, and three received education only through lectures or printed materials with no digital use recorded. Among healthcare providers, two reported using digital media, whereas four reported no experience with digital education.

### 3.2. Overview of Themes

Thematic analysis generated seven interrelated themes describing the experiences, barriers, and educational needs of adults with T2DM and healthcare providers in relation to diabetes self‐management and the potential role of immersive educational support. As summarized in Table 2, these themes included: (1) Psychological experiences in facing and managing diabetes; (2) motivation and adherence in diabetes self‐management; (3) adaptation of physical activity based on bodily condition and capacity; (4) challenges of dietary management in everyday life; (5) limitations and gaps in current diabetes education; (6) educational needs supporting diabetes self‐management; and (7) VR as an alternative medium for diabetes education. Together, these themes illustrate that diabetes self‐management was experienced not merely as a clinical or behavioral task, but as an emotionally, socially, and practically demanding process embedded in everyday life.

**TABLE 2:** Summary of themes and subthemes related to user needs for VR‐assisted diabetes self‐management support.

No	Theme	Subthemes	Summary of findings
1	Psychological experiences in facing and managing diabetes	Emotional reactions and disease recognition; Psychological burden of ongoing self‐management; Gradual acceptance and adaptation	Participants described diabetes as emotionally disruptive, especially at diagnosis, with fear, confusion, worry, and psychological fatigue emerging over time. Although some gradually adapted, self‐management remained mentally demanding and emotionally burdensome.
2	Motivation and adherence in diabetes self‐management	Internal and external sources of motivation; Behavioral challenges in maintaining adherence	Motivation was shaped by both self‐driven intentions and family support. However, adherence was often challenged by forgetfulness, boredom, low discipline, fluctuating motivation, and difficulty sustaining long‐term routines.
3	Adaptation of physical activity based on bodily condition and capacity	Physical activity as a practical and flexible health behavior; Adjustment to physical limitations; Fatigue, restriction, and the dilemma of activity	Physical activity was understood as routine, adaptable movement rather than formal exercise. Participants emphasized the need to adjust activity according to fatigue, bodily condition, and physical tolerance, often balancing perceived benefit with fear of overexertion.
4	Challenges of dietary management in everyday life	Diet was important but difficult; Confusion about food choices and portion sizes; Social pressures and dietary nonadherence	Although diet was seen as central to diabetes management, participants struggled with portion size, food selection, and maintaining control in daily and social contexts. Dietary behavior was shaped not only by knowledge, but also by habit, uncertainty, and social expectations.
5	Limitations and gaps in current diabetes education	Educational materials were often difficult to understand; Lack of specificity and practical applicability; Limited continuity and reinforcement	Existing diabetes education was often perceived as overly medical, too general, and difficult to apply in real life. Participants reported that written and verbal education often lacked practical guidance, relatable examples, and follow‐up reinforcement.
6	Educational needs supporting diabetes self‐management	Need for simple and practical education; Need for visual examples; Need for repeatable and confidence‐building support	Participants expressed a strong preference for education that was simple, visual, and easy to revisit. They valued examples that demonstrated movement, food portions, and routines in ways that were concrete, easy to imagine, and directly relevant to daily life.
7	Virtual Reality as an alternative educational medium	Expectations regarding VR design and function; Perceived educational value of immersive media; Concerns about usability and accessibility	VR was perceived as a potentially useful educational medium when it could provide realistic demonstrations, practical guidance, and context‐based learning. However, participants also emphasized that any immersive technology should remain simple, understandable, and easy to use.

### 3.3. Theme 1. Psychological Experiences in Facing and Managing Diabetes

#### 3.3.1. Emotional Reactions and Disease Recognition

Participants described their early experiences of diabetes through bodily symptoms such as frequent thirst, frequent urination, fatigue, blurred vision, itching, and weight loss. These symptoms often marked the first point at which they began to recognize that something was wrong, although many initially interpreted them as ordinary tiredness or temporary physical weakness.

One participant explained:

“At first, I often felt thirsty, urinated frequently, and got tired easily. I thought it was just ordinary fatigue. But after I checked at the health center, it turned out that my blood sugar was high.” (P3)

Recognition of the disease was frequently accompanied by fear, especially fear of uncontrolled blood sugar, sudden physical deterioration, and long‐term complications such as wounds or kidney problems. These concerns were often reinforced by stories, beliefs, and warnings from people around them. Some participants also described shock, confusion, sadness, and difficulty accepting the diagnosis, suggesting that the onset of diabetes was experienced as a psychologically disruptive event rather than simply a medical condition.

#### 3.3.2. Psychological Burden of Ongoing Self‐Management

Beyond the initial diagnosis, participants described a continuing psychological burden associated with long‐term self‐management. This burden was characterized by boredom, emotional fatigue, and mental exhaustion, particularly in relation to the ongoing need to regulate food, physical activity, medication, and blood glucose levels.

Participants often referred to diabetes management as tiring because it involved “too many rules” and required continuous self‐restraint. One participant stated:

“Often I get bored, sometimes tired, sometimes tempted to eat.” (P4)

This psychological fatigue was not only behavioral but also cognitive, as participants described constantly thinking about what to eat, what to avoid, and whether they were managing their condition correctly. These accounts indicate that diabetes self‐management was experienced as mentally demanding and emotionally draining over time.

#### 3.3.3. Gradual Acceptance and Adaptation

Despite these emotional and psychological difficulties, participants also described a gradual process of acceptance and adjustment. Over time, some began to integrate diabetes into their daily lives and developed more adaptive coping strategies. This process often involved learning from healthcare providers, asking questions, observing bodily responses, and gradually modifying habits such as medication‐taking and dietary regulation.

One participant reflected:

“At first it felt like a burden, very heavy, but now I am starting to accept it as part of my life.” (P1)

This suggests that living with diabetes involved not only distress and burden, but also a process of adaptation in which participants attempted to regain control and make the condition manageable within the realities of daily life.

### 3.4. Theme 2. Motivation and Adherence in Diabetes Self‐Management

#### 3.4.1. Internal and External Sources of Motivation

Participants described motivation as emerging from both internal and external sources. Family support was frequently identified as an important external motivator, particularly through reminders, emotional concern, and everyday supervision. Several participants emphasized that family members helped them remain aware of their condition and encouraged them to reduce unhealthy eating habits or maintain treatment routines.

One participant explained:

“They are the ones who remind me about my illness… my relatives tell me we should eat only a little.” (P5)

Internal motivation was also evident in participants’ desire to remain healthy, avoid complications, and preserve quality of life. Some participants expressed a strong wish to “live longer,” remain functional, or stay well enough to continue fulfilling family and personal roles. These findings suggest that motivation was not static but emerged from a combination of self‐preservation, emotional meaning, and social support.

#### 3.4.2. Behavioral Challenges in Maintaining Adherence

Although motivation was present, participants also described substantial behavioral barriers to adherence, particularly in relation to medication‐taking and maintaining consistency over time. These challenges included forgetfulness, boredom, low discipline, fluctuating awareness, and the persistence of old habits.

Healthcare providers also recognized that nonadherence often occurred when patients felt physically “fine” and therefore underestimated the seriousness of diabetes. One provider explained:

“The main problem is orderliness, regularity, and discipline in taking medication. Patients often feel they are fine, but when their sugar is checked, it is already 300–400.” (P13)

Participants also described stopping medication, forgetting to bring medicines, doubting whether medication was necessary, or becoming tired of taking it continuously. These accounts indicate that adherence was not solely determined by knowledge or intention, but was strongly influenced by routine disruption, emotional fatigue, and inconsistent self‐regulation.

### 3.5. Theme 3. Adaptation of Physical Activity Based on Bodily Condition and Capacity

#### 3.5.1. Physical Activity as a Practical and Flexible Health Behavior

Participants generally viewed physical activity as an important component of diabetes management, although it was rarely conceptualized as formal exercise alone. Instead, physical activity was often understood as routine movement embedded in daily life, such as walking, cleaning the house, stretching, or joining diabetes exercise sessions.

Many participants believed that activity did not need to be strenuous to be beneficial. As one participant stated:

“It does not have to be heavy exercise, as long as we stay active.” (P4)

Physical activity was perceived positively, particularly when it made the body feel lighter or was associated with improved blood sugar control. However, these benefits were usually described in practical rather than biomedical terms.

#### 3.5.2. Adjustment to Physical Limitations

Participants emphasized that physical activity had to be adapted to bodily condition and capacity. Rather than following rigid exercise prescriptions, they preferred forms of movement they perceived as realistic, tolerable, and safe. Activities such as walking, sweeping, stretching, and light household tasks were frequently mentioned as acceptable and sustainable.

One participant noted:

“Sometimes I get tired quickly, so if I’m advised to do something, I still have to adjust it to my body condition.” (P1)

These accounts suggest that participants approached physical activity through a lens of bodily negotiation, weighing perceived benefit against physical tolerance and safety.

#### 3.5.3. Fatigue, Restriction, and the Dilemma of Activity

Despite recognizing the value of physical activity, participants also described significant fatigue, bodily weakness, and restriction, which made adherence difficult. Several participants expressed a tension between wanting to be compliant and feeling physically unable to maintain activity consistently.

One participant described this as a dilemma:

“If I cannot stay active, I feel guilty and worried that my condition will get worse. But on the other hand, I know forcing myself can also be dangerous.” (P1)

These findings indicate that physical activity was not simply a matter of willingness, but was shaped by fluctuating physical capacity, energy, and perceived vulnerability.

### 3.6. Theme 4. Challenges of Dietary Management in Everyday Life

#### 3.6.1. Diet as Important but Difficult

Participants consistently acknowledged diet as a central part of diabetes management and believed that food choices strongly influenced their health. However, dietary regulation was also described as one of the most difficult aspects of living with diabetes.

Participants commonly described diet as important but burdensome, especially because it required restricting favorite foods and making repeated daily decisions. One participant stated:

“Diet is very important to keep blood sugar stable.” (P3)

At the same time, dietary management was often linked to broader ideas of living “more regularly” or “more healthily,” suggesting that diet was understood not only as food restriction but also as a lifestyle adjustment.

#### 3.6.2. Confusion About Food Choices and Portion Sizes

A major challenge involved uncertainty about what to eat, how much to eat, and how to interpret dietary advice in daily life. Participants described continued consumption of white rice, sweet foods, fried foods, and coconut‐based dishes, even while trying to reduce or control intake.

One of the most prominent concerns was the lack of clarity around portion size. Participants repeatedly reported confusion about what “moderate” or “sufficient” intake actually meant in practice. One participant explained:

“There is no really clear measure. I’m told to eat enough, but what does ‘enough’ actually mean?” (P1)

This suggests that dietary advice was often understood in principle but remained difficult to operationalize in everyday eating situations.

#### 3.6.3. Social Pressures and Dietary Nonadherence

Participants also described the influence of social situations, especially family gatherings, celebrations, and community events, as important barriers to dietary adherence. In these settings, refusing food was often experienced as socially uncomfortable or disrespectful.

One participant reflected:

“I was afraid of being seen as disrespectful, so I still ate even though I knew I should limit it.” (P4)

These findings highlight that dietary management was not only an individual challenge but also a socially negotiated behavior shaped by norms of hospitality, belonging, and interpersonal expectation.

### 3.7. Theme 5. Limitations and Gaps in Current Diabetes Education

#### 3.7.1. Educational Materials Were Often Difficult to Understand

Participants described several limitations in the educational materials they had received. A common concern was that information was often presented in medicalized language, with terminology that felt too technical, abstract, or difficult to understand. Written materials such as leaflets and brochures were also perceived as less helpful when they contained too much text or were visually tiring to read.

One participant stated:

“Sometimes the language is too medical, so I have to think very hard just to understand it.” (P1)

Participants also felt that educational content tended to be too general and insufficiently tailored to their individual circumstances, food habits, or day‐to‐day realities.

#### 3.7.2. Difficulty Translating Advice Into Practice

A recurrent problem was the gap between theoretical explanation and practical implementation. Although participants often understood the advice at the time it was given, many found it difficult to apply in real life, especially when faced with food choices, inconsistent routines, or uncertainty about what was “correct.”

One participant described this clearly:

“When it is explained, it seems easy, but when I try to do it in daily life, it turns out to be difficult.” (P1)

Participants also reported that important practical details, such as amount, frequency, duration, and specific examples, were often insufficiently explained, leaving them uncertain about how to implement recommendations confidently.

#### 3.7.3. Education Delivery Was Limited in Continuity and Format

Although participants received education from various healthcare professionals, including nurses, doctors, nutrition staff, and midwives, they often described the educational process as brief, occasional, and lacking follow‐up. Education was commonly delivered during clinic visits or after routine activities, and was often not reinforced over time.

One participant explained:

“Usually it is only once in a while during check‐ups. Outside of that, there is rarely any follow‐up education.” (P4)

While some participants mentioned receiving leaflets, posters, or videos, they also expressed a need for more engaging and interactive formats that could support ongoing understanding and recall.

### 3.8. Theme 6. Educational Needs Supporting Diabetes Self‐Management

#### 3.8.1. Need for Simple, Visual, and Practical Education

Participants expressed a clear preference for educational support that was simple, visual, and easy to apply. Visual examples, particularly videos, images, and demonstrations, were perceived as much easier to understand than text‐heavy or abstract explanations. These formats were especially valued for explaining movement, food portions, and daily routines.

One participant noted:

It is easier to understand when I can see it directly, especially through video.” (P5)

Participants also emphasized the need for education that was delivered in simple language, broken down into manageable steps, and directly connected to practical decision‐making in everyday life.

#### 3.8.2. Need for Accessible and Reassuring Educational Support

Participants wanted educational materials that could be accessed repeatedly and independently, especially because they often forgot instructions or needed to revisit explanations after leaving the clinic. Repetition was seen as important for confidence and retention.

At the same time, participants expressed a need for educational support that felt safe, nonjudgmental, and reassuring, allowing them to learn without fear of being blamed or misunderstood. These findings suggest that effective education must not only provide information but also create a sense of confidence, clarity, and emotional safety for users.

### 3.9. Theme 7. VR as an Alternative Medium for Diabetes Education

#### 3.9.1. Expectations for the Design and Function of VR‐Based Education

Participants and healthcare providers generally viewed VR as a potentially useful and interesting educational tool, particularly if it could provide clear examples, realistic demonstrations, and practical guidance relevant to everyday diabetes management. When discussing the potential use of visual and simulated educational content, participants generally viewed representations of food, portion sizes, and physical activity as potentially helpful for making diabetes self‐management guidance more concrete and understandable. Beyond these prompted examples, participants also described additional expectations for VR‐based education, including reminders, step‐by‐step instructions, familiar examples, and practical scenarios grounded in everyday routines. They further emphasized that any VR‐based intervention should be simple, relevant to daily life, and easy to understand. These accounts suggest that the perceived value of VR was linked not to novelty alone, but to its potential to make diabetes education more concrete and relatable.

#### 3.9.2. Concerns About Complexity and Usability

At the same time, participants expressed concerns that VR might be too complicated, unfamiliar, or difficult to use, especially for individuals with limited technological experience. Some worried about needing assistance, misunderstanding how to operate the system, or finding it too burdensome to use regularly.

These concerns highlight that while immersive educational tools may be attractive, their usefulness depends heavily on simplicity, accessibility, and ease of use, particularly in populations who may not be accustomed to advanced digital technologies.

#### 3.9.3. Recommendations for Future Development

Participants also provided suggestions for future development, emphasizing that educational media should include dietary guidance, medication routines, safe movement, stress management, culturally familiar examples, and practical strategies for everyday situations. They also expressed the importance of communication that is supportive, gradual, and easy to follow.

Taken together, these findings suggest that the acceptability of VR‐based diabetes education depends on its ability to address real‐world user needs rather than simply introducing technological novelty.

## 4. Discussion

This study explored the psychological, behavioral, and educational needs of adults with T2DM and healthcare providers to inform the development of VR‐assisted self‐management support. The findings demonstrate that diabetes self‐management is experienced not simply as a set of medical instructions, but as a complex and emotionally demanding process shaped by psychological burden, behavioral inconsistency, physical limitation, social context, and educational mismatch. This perspective is consistent with evidence showing that diabetes self‐management is deeply embedded in patients’ lived experiences and everyday contexts rather than being limited to compliance with medical recommendations [33]. Furthermore, the implementation of self‐management interventions is strongly influenced by contextual factors at the patient, professional, and interaction levels, highlighting the importance of tailoring support to real‐life conditions [34]. Collectively, these findings suggest that current diabetes education may not sufficiently address the lived realities through which self‐management decisions are made, thereby reinforcing the need for more contextual, user‐centered, and experientially meaningful educational approaches [35].

One of the central findings of this study is that living with T2DM is accompanied by substantial psychological burden, particularly during diagnosis and throughout long‐term disease management. Participants described fear, confusion, emotional distress, guilt, and fatigue in relation to both the illness itself and the continuous demands of self‐regulation. These findings align with the concept of diabetes distress, which reflects the emotional burden associated with managing the disease and includes feelings such as anxiety, guilt, and concern about self‐management demands [36]. In addition, broader evidence highlights that individuals with diabetes frequently experience significant psychosocial burdens and unmet emotional needs that are not adequately addressed in routine care [37]. Self‐management is also associated with self‐regulatory fatigue, where the continuous effort required to maintain health behaviors can reduce adherence and negatively affect outcomes.

In this study, psychological experiences were not peripheral to self‐management, but deeply embedded in how participants interpreted symptoms, responded to medical advice, and sustained or failed to sustain behavioral change. This reinforces the understanding that diabetes management is closely intertwined with emotional adaptation and self‐regulatory processes rather than being purely a matter of information or instruction. Therefore, diabetes education should move beyond knowledge transmission and explicitly address emotional responses, uncertainty, and the psychological work of living with a chronic condition.

The findings indicate that motivation and adherence in T2DM self‐management are dynamic and context‐dependent, shaped by both internal intentions and external reinforcement. Although participants expressed a strong desire to remain healthy and avoid complications, adherence was frequently disrupted by boredom, forgetfulness, fluctuating discipline, and the challenge of sustaining long‐term routines. These patterns are consistent with qualitative evidence showing that adherence is influenced by interacting psychological, sociocultural, and practical factors rather than motivation alone [38]. Family support emerged as an important source of motivation, highlighting that self‐management is socially embedded, with family members often providing encouragement, reminders, and practical assistance [39]. However, persistent behavioral barriers suggest that knowledge and motivation alone are insufficient when educational support fails to translate into sustainable daily action, particularly when information is inconsistent or poorly aligned with patients’ lived realities [40].

Another key finding relates to the embodied and adaptive nature of physical activity. Participants did not interpret physical activity through standardized or prescriptive targets, but rather through bodily tolerance, fatigue, energy, and daily practicality. This aligns with evidence indicating that engagement in physical activity is shaped by self‐efficacy, physical capacity, and the ability to sustain behavior over time [41], as well as by competing physiological and contextual demands in everyday life [42]. These findings reveal a disconnect between clinical recommendations and real‐world implementation, suggesting that educational approaches should move beyond prescriptive advice toward supporting individuals in interpreting what is safe, realistic, and meaningful within their own contexts. This has important implications for immersive educational design, where scenario‐based learning may facilitate adaptive decision‐making and context‐sensitive self‐management.

Dietary management emerged as one of the most difficult aspects of self‐management, not because participants lacked awareness, but because they struggled to translate general advice into practical eating behaviors within family and social contexts. Participants described uncertainty about portion sizes, appropriate intake, and maintaining dietary control in everyday situations. These findings suggest that dietary behavior is shaped not only by knowledge, but also by cultural norms, social expectations, and difficulty interpreting food‐related guidance. Educational interventions should therefore provide more contextualized examples, culturally familiar foods, and practical portion guidance [43, 44]. This study also highlights persistent limitations in current diabetes education, particularly in terms of language, practicality, and relevance to daily life. Participants often perceived existing education as too general, overly medical, and difficult to apply outside the clinical setting. This finding indicates that the challenge lies not only in access to education, but also in the mismatch between how education is delivered and how self‐management is actually practiced in real‐world contexts.

Closely related to this, participants expressed a strong need for educational support that is simple, visual, repeatable, and directly applicable to daily life, emphasizing formats that could demonstrate movement, clarify food portions, and provide examples that were easy to understand and revisit. These preferences suggest that patients do not merely need more information, but rather educational delivery that reduces ambiguity, supports recall, and strengthens confidence in everyday decision‐making. This is consistent with evidence showing that effective diabetes‐related digital interventions depend heavily on usability, accessibility, relevance, and user‐centered tailoring, particularly in populations with varying levels of digital health literacy [19, 45–47]. Within this context, the findings provide a strong rationale for considering VR as a potentially valuable educational modality. Importantly, the perceived value of VR was not based on technological novelty alone, but on its potential to make difficult or abstract self‐management recommendations more understandable, visible, and actionable through realistic, step‐by‐step simulation of daily practices. At the same time, the educational value of immersive technology is likely to depend less on immersion as spectacle and more on whether it is designed in ways that are simple, low‐burden, culturally appropriate, and aligned with users’ real‐world needs and capabilities, consistent with broader evidence on user‐centered digital health design and engagement in diabetes care [48, 49].

At the same time, this study highlights important design considerations and cautions for the use of immersive technologies in diabetes care. Participants expressed concern that advanced technologies may be confusing, overly complicated, or difficult to use independently, particularly for individuals with limited digital familiarity. This finding is important because it cautions against assuming that technological sophistication automatically translates into educational effectiveness. Instead, the success of VR‐based interventions will likely depend on whether they are designed to be simple, usable, low‐burden, and responsive to users’ everyday needs and capabilities [50, 51]. These concerns are further reinforced by evidence showing that digital health literacy among adults with chronic conditions is shaped by accessibility, digital adaptation skills, cognitive understanding, and social support, all of which may influence how effectively patients engage with technology‐based education [18, 52]. Taken together, these findings underscore the importance of user‐centered design, particularly in settings where digital literacy, health literacy, and resource accessibility may vary substantially [53]. These considerations may be particularly important for rural or geographically underserved populations, where limited access to digital infrastructure, healthcare services, and ongoing diabetes education may create additional barriers to technology‐supported self‐management. In the present sample, digital familiarity ranged from no digital use to regular use of digital media (Table 1), and concerns about complexity were raised even though VR was generally viewed as potentially useful. This suggests that digital familiarity should be treated as a design consideration rather than an assumption. VR‐assisted education should therefore include simple navigation, orientation from healthcare providers during initial use, and options for supported use within primary care services, rather than presuming independent use.

### 4.1. Future Implications for VR‐Assisted Diabetes Education

More broadly, this study provides a user‐informed foundation for the development of VR‐assisted diabetes self‐management support. The novelty of this work lies not in assuming that immersive technology alone can resolve adherence challenges, but in identifying the specific psychological, behavioral, and educational needs that such interventions should be designed to address. These include emotional reassurance, practical dietary guidance, realistic adaptation of physical activity, repeatable and accessible learning, and context‐sensitive support for daily decision‐making. Because this study represents the user‐needs assessment stage rather than the evaluation of a finalized VR prototype, these findings should be interpreted as preliminary design requirements for subsequent development. Based on these requirements, the proposed VR intervention could incorporate realistic everyday scenarios in which users can visualize and rehearse self‐management decisions, supported by simple navigation, step‐by‐step guidance, familiar examples, and manageable amounts of information. The design should also enable users to revisit relevant educational scenarios at their own pace while minimizing unnecessary technological complexity and user burden. These design considerations are consistent with broader recommendations for healthcare VR development, which emphasize usability, intuitive interaction, alignment between technological features and educational objectives, user capability, and iterative evaluation involving end users and healthcare professionals [54, 55]. In this way, the findings reposition technology not as the innovation itself, but as a delivery medium for more meaningful, contextually grounded, and behaviorally usable education, consistent with broader principles of user‐centered digital health design.

An important question is whether VR is genuinely needed by this population. Participants described current education as brief, occasional, text‐heavy, and difficult to apply, consistent with reports from Indonesian primary care that education is limited and not tailored to literacy levels or cultural context. They preferred simple, visual, repeatable, and practical formats, and both patients and providers viewed VR as potentially useful for concrete demonstrations of food portions, physical activity, and daily routines, while raising concerns about complexity and unfamiliarity; limited smartphone access has also been reported as a barrier in Indonesian primary care [14]. These findings support the need for more practical, visual education and indicate that VR is a plausible way to meet it, but they do not establish that it is necessary or superior to alternatives such as video‐based education or caregiver‐supported counselling, which should be examined through prototype testing and comparison with conventional or simpler digital formats.

Based on these needs, the envisioned VR program would be organized around visualization of food portions using culturally familiar foods, physical activity simulations adjusted to bodily capacity, step‐by‐step medication routines, and supportive, nonjudgmental communication that acknowledges emotional burden. As outlined in the Introduction, its proximal outcomes (understanding and self‐efficacy) address the difficulty participants described in translating advice into practice, while behaviors, glycemic control, and distress are longer‐term outcomes. Given cost, accessibility, and digital familiarity, hardware, interaction design, and usability will be refined through co‐design and feasibility testing. These outcomes are hypotheses for future evaluation, not findings of this study.

Sustainability and real‐world impact cannot be inferred from a user‐needs study. Evidence on VR‐based interventions in T2DM remains modest: a systematic review found no superior glycemic or behavioral efficacy compared with traditional approaches, although VR offered advantages in adherence and accessibility [24], and a 52‐week randomized trial of a 3D virtual environment reported greater weight loss than a control website but no significant differences in most other outcomes [56]. Digital health solutions for T2DM also face barriers such as inadequate personalization, cultural and language mismatch, and difficulty sustaining engagement [49]. Development should therefore plan from the outset for ease of use, culturally and linguistically appropriate content, affordable and maintainable delivery, and the ability to revisit content. As this evidence comes largely from other health‐system contexts, its applicability to Indonesian primary care is uncertain; these elements should be examined in feasibility and effectiveness studies with long‐term follow‐up and implementation outcomes before any claims about patient impact are made.

Traditional approaches, including health education and caregiver or family support, can be highly effective, and our findings do not suggest replacing them. Participants described learning from healthcare providers and described family members as a source of encouragement, reminders, and practical help [39]. This is consistent with a complementary role for VR [24]. The limitations participants described concerned how education is delivered (brief, text‐heavy, lacking follow‐up) rather than its value. We therefore position the envisioned VR program as a complement that extends provider‐delivered education, for example, by letting patients revisit demonstrations after clinic visits. Family members could join sessions, but because family involvement can be helpful or unhelpful, caregivers would also need guidance [57]. Whether this combined approach adds value over conventional or family‐supported care should be tested in comparative studies.

Without proper monitoring and guidance, educational technology may not translate into sustained benefit. Participants voiced this concern, worrying about needing help to operate VR, forgetting instructions after leaving the clinic, and lacking follow‐up. Consistent with evidence that users need adequate training for immersive and digital health tools [23] and that VR implementation protocols remain to be explored [24], the envisioned program is guided rather than self‐directed: (1) Providers, such as primary care nurses, orient patients and supervise first sessions at the health center; (2) use is monitored through application‐logged indicators (sessions completed, time spent, content revisited) and brief provider check‐ins, since digital tools often struggle to sustain engagement [49]; (3) providers reinforce key messages and link VR sessions to existing education and follow‐up services; and (4) comfort and safety, such as session length and dizziness, are reviewed each session, with difficulties prompting additional support. These are design proposals to be tested in feasibility studies, including monitoring of engagement and drop‐out.

The role of government is central to whether VR‐assisted diabetes education can be provided at scale, and policies differ across countries. In Indonesia, the Ministry of Health leads a national digital health transformation with a government‐managed health services platform, a focus on Puskesmas, telemedicine expansion at first‐level facilities, regulatory sandboxes, and strengthened digital literacy of the health workforce [20]. However, this blueprint does not specifically address immersive technologies, and we are not aware of an established policy or financing mechanism for VR‐based diabetes services; this study therefore does not assume such services are currently government‐provided. If a VR program proves feasible and effective, government support would be relevant for regulatory and quality standards and integration into existing primary care education such as Prolanis [20], training and supervision of providers, and financing and equipment for Puskesmas. These considerations are tentative, specific to Indonesia, and should be examined with policymakers in future feasibility and implementation research.

The findings have several practical implications for diabetes education and nursing practice. First, educational interventions for adults with T2DM should be designed to address not only biomedical knowledge but also emotional burden, uncertainty, and the practical realities of daily self‐management. Second, diabetes education should be delivered using language, examples, and media formats that are accessible, relevant, and easy to revisit over time. Third, nurses and other healthcare providers may benefit from educational tools that help bridge the gap between standard advice and patients’ lived implementation challenges. Finally, immersive digital tools such as VR may have value when used not as stand‐alone technological solutions, but as supportive educational platforms that are co‐designed around real user needs. This approach is consistent with emerging applications of immersive technology in diabetes and other healthcare contexts, where interventions have been designed around user‐centered development, realistic learning experiences, and iterative evaluation. For example, Bishop et al. (2023) used a human‐centered design approach to develop and refine an avatar‐based diabetes education program through user input and prototype testing [25]. Similarly, Waite et al. (2026) evaluated an immersive 3D VR‐CAVE diabetes education environment and elicited users’ experiences of specific design elements and recommendations for future development [26]. In another healthcare context, the Tandem VR approach provided synchronized immersive experiences for hospice patients and their caregivers, illustrating how VR can be tailored to users’ personal and relational contexts [48, 58]. Collectively, these studies support the importance of designing immersive health education around users’ needs, real‐life contexts, and iterative feedback rather than technological novelty alone [49].

### 4.2. Strengths and Limitations

A key strength of this study is that it incorporates the perspectives of both adults with T2DM and healthcare providers, allowing a richer understanding of self‐management needs from both lived and professional standpoints. In addition, the study was conducted in a primary care and community‐based context, enhancing the practical relevance of the findings for frontline diabetes education.

However, several limitations should be acknowledged. First, the study was conducted in a specific geographic and health service context, which may limit transferability. Second, findings are based on self‐reported experiences and perceptions, which may be influenced by recall and social desirability. Third, because the study focused on needs exploration rather than intervention testing, the feasibility, acceptability, effectiveness, long‐term sustainability, cost, and integration into routine care of VR‐assisted education were not assessed and should be examined in future trials. Fourth, participants’ digital familiarity varied, and VR was introduced through a brief verbal explanation, without a prototype, and without prior technology experience as an eligibility criterion; views of participants with limited digital familiarity should therefore be read as expectations, not an experience‐based evaluation. Fifth, the provider group was small (five nurses, one physician), and family members or caregivers, nutrition staff, and older adults (participants with T2DM were aged 26–54 years) were not included, so their perspectives remain to be explored.

## 5. Conclusion

Adults with T2DM experience self‐management as a complex process shaped by emotional burden, behavioral challenges, social context, and limitations in existing educational support. This study shows that current diabetes education may not fully address the practical and contextual realities through which patients make everyday decisions about diet, physical activity, and routine self‐care. By identifying these user needs from both patient and healthcare provider perspectives, this study provides an important foundation for the development of VR‐assisted diabetes self‐management support. Future interventions should prioritize simplicity, usability, contextual relevance, and behavioral meaningfulness to ensure that immersive technologies function not merely as novel tools, but as accessible and effective supports for daily diabetes self‐management.

## Funding

This research was funded by the Indonesian Endowment Fund for Education (LPDP) on behalf of the Indonesian Ministry of Higher Education, Science and Technology and managed under the EQUITY Program (Contract No. 4307/B3/DT.03.08/2025 and 47103/UN4.1/PM.01.01/2025).

## Conflicts of Interest

The authors declare no conflicts of interest.

## Supporting Information

Additional supporting information can be found online in the Supporting Information section.

## Supporting information


**Supporting Information** Supporting Table S1. Semi‐structured interview guides for adults with T2DM and healthcare providers

## Data Availability

The data that support the findings of this study are available from the corresponding author upon reasonable request.
