# Supplementary material for: Toward Immersive Diabetes Education: Understanding User Needs for Virtual Reality–Assisted Self‐Management Support in Adults With Type 2 Diabetes Mellitus
Source: Nurs Res Pract. 2026 Nov 6;2026:4385505. doi: 10.1155/nrp/4385505 (PMC13741207; doi:10.1155/nrp/4385505)
Supplement: Supplementary file 1 [file NRP-2026-4385505-s001.docx]

Supplementary Table S1. Semi-structured interview guides for adults with T2DM and healthcare providers

| **Domain** | **Main questions** | **Probes** |
| --- | --- | --- |
| **Interview guide for adults with Type 2 Diabetes Mellitus** | | |
| 1. General experience of managing diabetes | 1.1. Could you tell us about your experience since you were first diagnosed with diabetes? | What changes have been most noticeable in your daily life? |
|  | 1.2. What role does diet play in managing your diabetes? | — |
|  | 1.3. What role does physical activity play in managing your diabetes? | — |
| 2. Experience with dietary education (3J: amount, type, and timing) | 2.1. What forms of dietary education have you received so far, and from whom? | — |
|  | 2.2. Which aspects of dietary education have been the most difficult for you to understand or apply? | Food portion sizes; food choices; meal timing; social situations such as family gatherings or eating out. |
|  | 2.3. Do you feel that the dietary education you have received is appropriate to your condition and everyday habits? | — |
| 3. Experience with physical activity education | 3.1. What types of physical activity have usually been recommended to you? | — |
|  | 3.2. What difficulties do you experience in performing physical activity regularly? | Fear; physical condition; lack of time; motivation; lack of clear examples. |
|  | 3.3. Do you think the physical activity education you have received is sufficiently clear, or is it still confusing? | — |
| 4. Experience with educational media | 4.1. What types of media have been used in the diabetes education you have received (e.g., leaflets, posters, videos, lectures, or applications)? | — |
|  | 4.2. Which educational media have been most helpful to you, and why? | — |
|  | 4.3. What are the limitations of the educational media you have received so far? | — |
| 5. Perceptions of technology-based and Virtual Reality education | 5.1. Have you ever used digital technology, such as applications or interactive videos, to learn about health? | — |
|  | 5.2. If an educational medium could realistically simulate food, portion sizes, and physical activity, do you think this would be helpful? Why or why not? | — |
|  | 5.3. What would you expect from a technology-based educational medium such as Virtual Reality? | — |
| 6. User needs and expectations | 6.1. If you could design an educational medium yourself, what features would you consider most important? | — |
|  | 6.2. What would make you feel comfortable or uncomfortable when using technology such as VR? | — |
|  | 6.3. In your opinion, how should educational media be designed so that they are genuinely helpful in everyday life? | — |
| **Interview guide for healthcare providers** | | |
| 1. Experience providing diabetes education | 1.1. What has been your experience in providing dietary and physical activity education to patients with T2DM? |  |
|  | 1.2. What challenges do you most frequently encounter during the educational process? |  |
| 2. Perceptions of patients’ understanding | 2.1. In your experience, which aspects of diabetes education are the most difficult for patients to understand? |  |
|  | 2.2. What factors most strongly influence the success of dietary and physical activity education? |  |
| 3. Educational media needs | 3.1. What educational media are currently available and used? |  |
|  | 3.2. What are the limitations of these educational media? |  |
| 4. Views on Virtual Reality | 4.1. What is your opinion about the use of Virtual Reality as a medium for diabetes education? |  |
|  | 4.2. What content do you consider most important to include in VR-based education on diet and physical activity? |  |
| 5. Recommendations for system development | 5.1. What features should be included to ensure that VR-based educational media are effective and safe for patients to use? |  |
|  | 5.2. What should be avoided so that the technology does not make diabetes education more difficult for patients? |  |
| Closing question | Is there anything else you would like to share regarding diabetes education or your expectations for future educational media? |  |
